# ﻿A new species of *Oecetis* McLachlan, 1877 (Trichoptera, Leptoceridae) and new distributional records of Trichoptera in the eastern Amazon

**DOI:** 10.3897/zookeys.1263.151613

**Published:** 2025-12-10

**Authors:** Fábio B. Quinteiro, Anne M. Costa, Gabriel Saldanha, Laisse Moura, Otávio T. Assunção, Leandro Juen

**Affiliations:** 1 Laboratório de Estudos Comparativos em Insetos, Universidade Federal do Pará (UFPA), Instituto de Estudos Costeiros (IECOS), 68600-000, Bragança, Pará, Brazil Universidade Federal do Pará Bragança Brazil; 2 Laboratório de Ecologia e Conservação, Universidade Federal do Pará (UFPA), Instituto de Ciências Biológicas (ICB), 66075-110, Belém, Pará, Brazil Universidade Federal do Pará Belém Brazil

**Keywords:** Aquatic insects, biodiversity, long-horned caddisflies, Neotropics, taxonomy

## Abstract

The Amazon is one of the most diverse biomes on Earth, and most of its area is in the territory of Brazil. Even though it harbors most of Earth’s diversity, species remain to be described. Therefore, knowing the biodiversity of the Amazon is of utmost importance, and this must be done quickly because the biome is severely under threaten by deforestation. Currently, the Brazilian Amazon has around 340 caddisfly species records, many endemic. To fill gaps in biodiversity knowledge of the Brazilian Amazon, we describe a new species of *Oecetis* and expand the known distributions of eight species of caddisflies. *Oecetis amplicauda***sp. nov.** can be distinguished from the other species in the *O. testacea* group by the cylindrical dorsal portion of tergum X, longer than the preanal appendage, and by the inferior appendage with a distinctly enlarged dorsal lobe with stout setae. Four other species of Trichoptera are recorded for the first time for Brazil: *Nectopsyche taleola* Flint, 1974, *Oecetis inflata* Flint, 1974, *Polyplectropus alienus* Bueno-Soria, 1990, and *Polyplectropus flintorum* Chamorro & Holzenthal, 2010. Three valid species are recorded for the first time in the state of Pará: *Marilia paraguassu* Rocha & Souza, 2018, *Nectopsyche splendida* (Navás, 1917), and *Polyplectropus rondoniensis* Chamorro & Holzenthal, 2010. These results highlight that some caddisfly taxa still lack thorough studies in critical biomes, such as the Amazon, and that imperative actions towards conserving these areas are necessary.

## ﻿Introduction

The Brazilian Amazon harbors almost 340 species of caddisflies (Santos et al. 2025). Its area encompasses more than 5 million km^2^ and covers nine states of the country (IBGE 2022). The biome can be subdivided into the Western and Eastern Amazon. Each of these subregions has significant differences in their topography and climatological patterns of seasonal precipitation along with specific socioeconomic and regional issues (Amanajás and Braga 2012). With more than 1.2 million square kilometers, Pará is the second largest state by area in the Brazilian Amazon and the largest in the Eastern Amazon (IBGE 2023). The state of Pará currently has 122 caddisfly species records, distributed among 10 families and 30 genera (Santos et al. 2025). Despite the recent increase in knowledge on trichopteran diversity in the Eastern Amazon, there are many more species in this region yet to be described (Santos et al. 2020, 2025).

Among the trichopteran family present in Pará, Hydropsychidae, Leptoceridae, and Hydroptilidae have the most species, representing more than 50% of local diversity (Santos et al. 2025). Nevertheless, families like Odontoceridae and Polycentropodidae are also representatives of the fauna and are abundantly collected.

Leptoceridae is the second largest family in Pará, with only 22 species (Santos et al. 2025). *Nectopsyche* Müller, 1879 and *Oecetis* McLachlan, 1877 are the most diverse genera, with 11 and eight species, respectively (Santos et al. 2025). *Oecetis* is a worldwide distributed genus, with almost 600 described species (Moura and Quinteiro 2023; Bonfá-Neto et al. 2024; Morse 2025), with 76 of them recorded in the Neotropics (Holzenthal and Calor 2017; Calor et al. 2023; Bonfá-Neto et al. 2024) and 36 of them recorded from Brazil (Santos et al. 2025). Adults of *Oecetis* can be diagnosed by the unbranched M vein on the forewings and the stout maxillary palpi (Quinteiro and Almeida 2021). Even though the genus is highly diverse, several species remain to be described (Quinteiro and Holzenthal 2017; Quinteiro and Almeida 2021).

To fill gaps in knowledge of species’ distribution and diversity in this biome, we describe a new species of *Oecetis* and expand the known distributions of seven species of caddisflies in the eastern Amazon.

## ﻿Methods

### ﻿Collection methods and description of the study area

This study was based on male specimens collected with light traps (see Calor and Mariano 2012, but adapted to LED lights) in various localities in the state of Pará. Specimens were preserved in 80% ethanol.

The new species was collected in the northeast of Pará (Bragança), and species that represented the new records were collected in the Belém metropolitan region (Barcarena), western Pará (Itaituba and Parque Nacional do Jamanxim - PARNA Jamanxim), and southeastern Pará (Altamira and Reserva Biológica Nascente da Serra do Cachimbo - REBIO). PARNA do Jamanxim was created to promote the sustainable use of forest resources and the protection of water resources and biodiversity (ICMBio 2010). The REBIO Serra do Cachimbo is a biological reserve created to preserve perennial springs from the Xingu and Tapajós river basins (ICMBio 2009).

The distribution map was created using QGIS v. 3.40.3 and Adobe Photoshop 2021.

### ﻿Identification and illustrations

Specimens were identified using a stereomicroscope to study morphological characteristics of wing venation and male genitalia. To study the structures of the genitalia, the abdomen was carefully removed and submitted to a clearing process using a solution of 85% lactic acid, as described in Blahnik et al. (2007). Specimens were identified using the primary literature. Illustrations were made with a microscope and an attached drawing tube. Photographs were taken using a Leica M205A stereomicroscope, with multifocus photo assembly. After the preparation of pencil templates, the figures were made using Adobe Illustrator (Adobe Inc.). Figures showing venation and genital homology hypothesis were also made in Adobe Illustrator. The genital color code is as follows: blue = segment IX, purple = preanal appendages, brown = tergum X, green = inferior appendages, and red = phallic apparatus (dark for phallobase; light for endotheca). Morphological terminology follows Quinteiro and Almeida (2021).

### ﻿Voucher specimens

Type material of the new species have been deposited in the Museu Paraense Emilio Goeldi (**MPEG**), Belém, Pará, Brazil, Museu de História Natural da Universidade Federal da Bahia (**UFBA**), and Museu Nacional do Rio de Janeiro (**MNRJ**). Non-type specimens have been deposited in Coleção Zoológica, Universidade Federal do Pará, campus Bragança (**CZBr**).

## ﻿Results

### ﻿ Oecetis amplicauda

Taxon classificationAnimalia

Quinteiro & Saldanha sp. nov.

9166155C-2F0F-5358-B870-0E788683B249

https://zoobank.org/368FBA72-634D-4D8D-93F4-51047B0DAEF4

Figs 1, 2, 3

#### Type locality.

Brazil, Pará: Bragança, Jiquiri, ramal Arauá, Sítio Cururutuia, 01°04'42.22"S, 46°44'14.58"W, alt 19 m.

#### Type material.

***Holotype***: Brazil • ♂; Pará, Bragança, Jiquiri, ramal Arauá, Sítio Cururutuia; 01°04'42.22"S, 46°44'14.58"W; alt 19 m; 16 Apr. 2021; light trap; F.B. Quinteiro & A.M. Costa leg.; MNRJ-ENT10-362. ***Paratypes***: #1 ♂; same data as for holotype; UFBA T1859 • 1 ♂; same as for holotype MPEG.HEX 05040018 • 1 ♂; same as for holotype, except 15 May 2021; MPEG.HEX 05040019 • 1 ♂; same as for holotype, except 11 Apr. 2022; MPEG.HEX 05040020.

#### Diagnosis.

Due to the honeycomb microstructure covering terga V–VIII, this species belongs to the *O. testacea* species group as defined by Quinteiro and Holzenthal (2017). It can be distinguished from other species in this group by having the dorsal portion of tergum X longer than the preanal appendage, cylindrical, and with an acute apex in dorsal view. Also, the inferior appendage has an enlarged basis and a rounded dorsal lobe which bears short, thick setae, and with an inward-directed apex.

#### Description.

**Figure 1.**
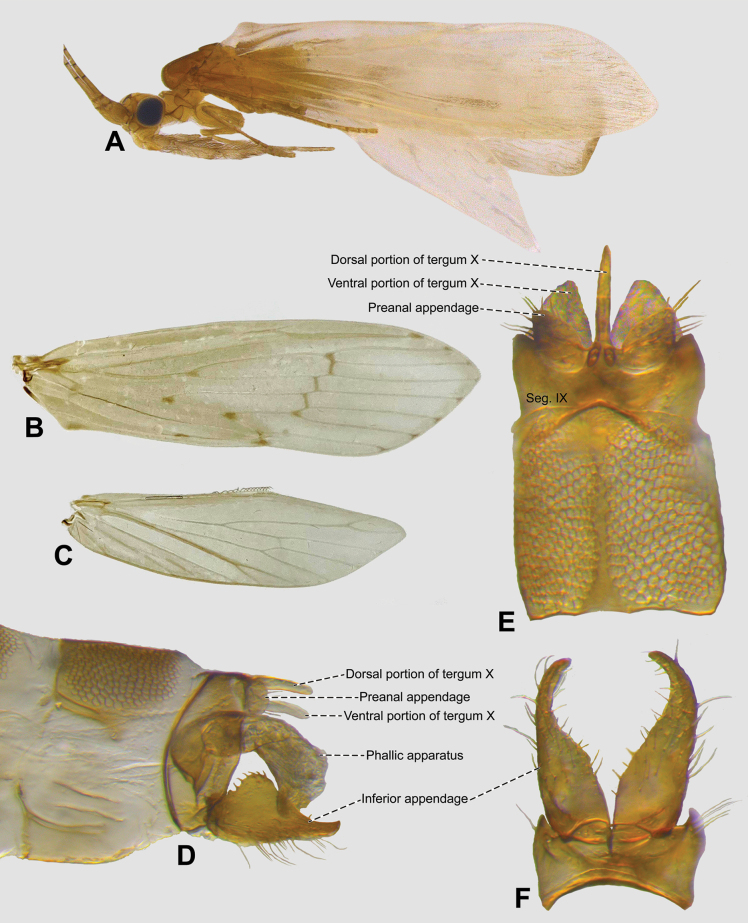
*Oecetis amplicauda* sp. nov., male holotype. **A.** Habitus, lateral view, wings; **B.** Forewing; **C.** Hindwing; **D–F.** Genitalia in lateral, dorsal and ventral views, respectively.

**Male.** General color yellowish brown in alcohol (Fig. 1A). Forewing length 5.0–5.9 mm, (*n* = 4), holotype 5.2 mm. Hindwing length 4.0–4.9 mm (*n* = 4), holotype 4.8 mm.

Head yellowish brown (Fig. 1A). Antennae three times length of forewing; scape stout; pedicel short. Anteromesal setal wart small, rounded; posterior setal wart rounded; posterolateral setal wart thin. Maxillary palps yellowish brown, 5-segmented (Fig. 1A); labial palps yellowish brown, 3-segmented (Fig. 1A).

**Figure 2.**
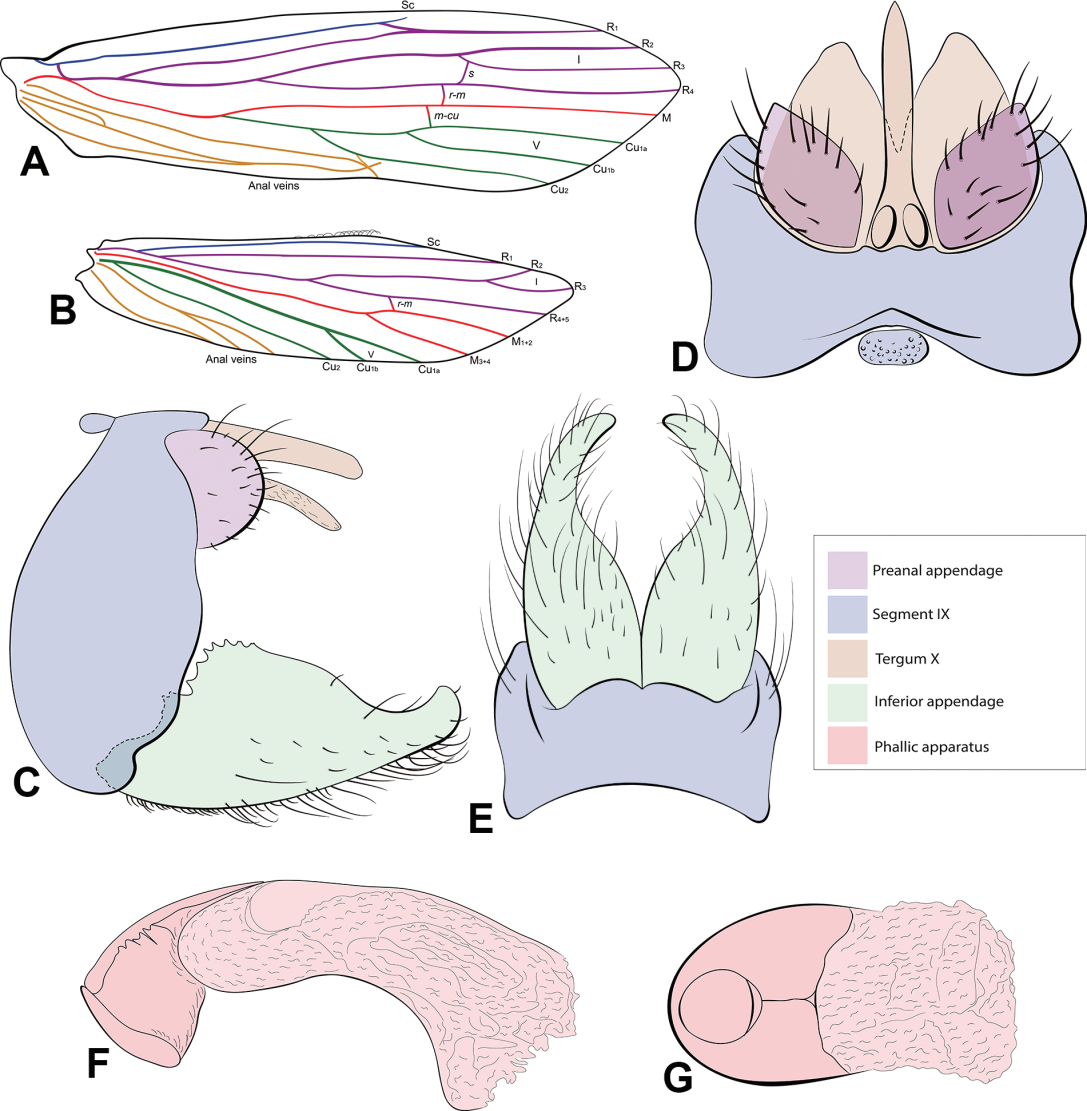
*Oecetis amplicauda* sp. nov., male. **A.** Forewing; **B.** Hindwing; **C–E.** Lateral, dorsal, and ventral views, respectively; **F.** Phallic apparatus, lateral view; **G.** Phallic apparatus, ventral view. Crossveins are represented in lowercase italicized letters and forks in Roman numerals.

Thorax. Pterothorax brown. Forewings yellowish brown (Fig. 1A, B); dark bands present on forewing over chord (*s*, *r-m*, *m-cu*); dark spots present on forks, junctions, and vein tips; colored setae absent; forks I and V present, both rooted; sectoral crossvein (*s*) distal of *r-m*; *m-cu* crossvein basal of *r-m* (Figs 1B, 2A). Hindwings yellowish brown; forks I and V present (Figs 1C, 2B). Legs yellowish brown. Tibial spur formula 1,2,2; foreleg tibial spur ½ length of mid- and hindleg spurs.

Abdomen. Reticular patches (honeycomb texture, Fig. 1D, E) on terga V–VIII. Acrotergite present between segments VIII and IX (Figs 1D, E, 2C, D). Segment IX short, medially enlarged and posterolateral process absent in lateral view (Figs 1D, 2C); wider than inferior appendages in ventral view, with anterior margin concave (Figs 1F, 2E). Preanal appendages short, apex rounded, basally broadened in lateral view, with apical setae (Figs 1D, 2C), ovate, with round base and acuminate apex in dorsal view (Figs 1E, 2D). Tergum X longer than preanal appendage, dorsoventrally divided in lateral view (Figs 1D, 2C); dorsal portion slightly longer than ventral, tapering apically, with apex acuminate, ventral portion mesally divided, basally broadened, apex rounded in dorsal view (Figs 1E, 2D). Inferior appendage 1-segmented, setose, basally enlarged; dorsal lobe enlarged, apex rounded, projecting dorsad, with stout, short setae in lateral view (Figs 1D, 2C); ventral lobe absent (Figs 1D–F, 2C–E); distal lobe concave, smoothly apically tapering, apex acuminate, slightly curved inward, obtuse angle formed by dorsal and distal lobes (Figs 1D–F, 2C–E). Phallic apparatus symmetrical, elongate, cylindrical, ventrally bent; phallobase basally large, sclerotized, slightly constricted subasally (Figs 1D, 2F, G); endotheca twice as long as phallobase and 1-lobed in lateral view (Figs 1D, 2F), same length in ventral view (Fig. 2G); phallic spines absent; phallotremal sclerite absent (Figs 1D, 2F, G).

Female and immatures. Unknown.

#### Etymology.

*amplio* (Latin) = enlarged; *cauda* (Latin) = tail, appendage. The species name is a reference to the enlarged inferior appendages, with a prominent dorsal lobe.

#### Distribution.

**Figure 3.**
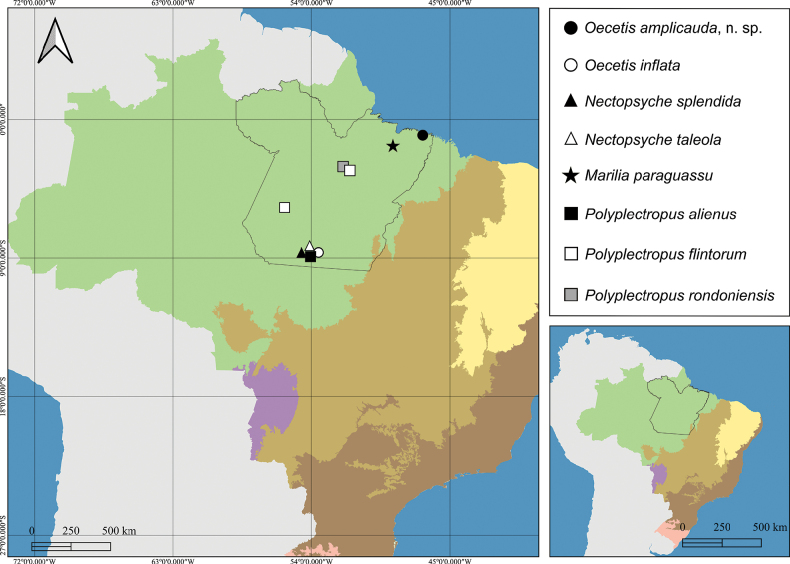
Distribution map of *Oecetis amplicauda* sp. nov. and new records of caddisflies in the state of Pará, Brazil. Colored areas represent biomes of Brazil: green = Amazon rainforest; light brown = Cerrado; purple = Pantanal wetland; dark brown = Atlantic Forest; yellow = Caatinga; light pink = Pampa.

Known only from the type locality in Pará state, Brazil (Fig. 3).

#### Taxonomic remarks.

*Oecetis amplicauda* sp. nov. belongs to the *O. testacea* group of Quinteiro and Holzenthal (2017) due to the honeycomb-like texture on the abdominal terga. It is the fourth species described, along with *O. iara* Henriques-Oliveira, Dumas & Nessimian, 2014, *O. plenuspinosa* Quinteiro & Holzenthal, 2017, *O. meronai* Gibon, 2019, and *O. ancorospina* Moura & Quinteiro, 2023. Among the *O. testacea*-group species, it is most similar to *O. ancorospina* Moura & Quinteiro, 2023. These two species share a rounded preanal appendage, a long, cylindrical dorsal portion of tergum X, and an elongate phallic apparatus. However, the new species has segment IX slightly medially enlarged in lateral view, with no anterior or posterolateral projections. In *O. ancorospina*, segment IX has a distinct projected anterolateral margin and presents a posterolateral projection. The inferior appendages in *O. amplicauda* sp. nov. are basally enlarged and with a distinct dorsal lobe, while in *O. ancorospina*, the inferior appendages are basally constricted. Also, the distal lobe of the inferior appendages in *O. ancorospina* is large, with a truncate apex, while in *O. amplicauda* sp. nov., they taper distally, with an acuminate apex, which is bent inwards. Additionally, *O. amplicauda* sp. nov. is the only known species in the *O. testacea*-group that has no phallotremal sclerite or phallic spine.

##### ﻿New records of caddisflies

###### ﻿Leptoceridae

Leptoceridae, also known as long-horned caddisflies, have a worldwide distribution and approximately 1,800 species and 47 genera (Holzenthal and Calor 2017). The family is divided into four subfamilies (Malm and Johanson 2011), of which Leptocerinae has the greatest diversity in Pará (Santos et al. 2025). Here, we present new data on three species in the state (Fig. 3).

### ﻿ Nectopsyche splendida

Taxon classificationAnimalia

(Navás, 1917)

D432D38E-65C4-5EA1-9A30-430EDE7E4F03

#### Literature records.

Navás 1917: 403 [type locality: Argentina, Santa Fe; collection Navás, now lost?; ♂; in *Leptocella*]. Schmid 1949: 386 [no ♂ in Navás collection]. Flint 1982: 60 [redescription; distribution]; Flint 1991: 72 [distribution]; Flint 1996:418 [distribution]; Almeida and Marinoni 2000: 349 [distribution; biology]; Muñoz-Quesada 2000: 279 [checklist]; Paprocki et al. 2004: 13 [checklist]; Paprocki and França 2014: 60 [checklist]; Quinteiro et al. 2014: 233 [distribution]; Desidério et al. 2017: 165 [distribution]; Holzenthal and Calor 2017: 329 [catalog]; Bonfá-Neto and Salles 2023: 5 [distribution].

#### Material examined.

Brazil • 1 ♂; Pará, Novo Progresso, REBIO Serra do Cachimbo; 08°44'19.41"S, 54°36'4.81"W; 10 May 2023; light trap; Leandro Juen exped; CZBr 000325 • 2 ♂; same collection data as for preceding; 08°42'33.73"S, 54°40'19.49"W; 11 May 2023; CZBr 000326.

#### Distribution.

Argentina, Bolivia, Brazil (Bahia, Espírito Santo, Maranhão, Minas Gerais, **Pará**, Paraná, Piauí, Roraima), Colombia, Ecuador, Guyana, Paraguay, Peru, Venezuela.

### ﻿ Nectopsyche taleola

Taxon classificationAnimalia

Flint, 1974

7D481350-DF58-5B4E-AFF9-C5E259D7DCB3

#### Literature records.

Flint 1974: 134 [Type locality: Suriname, Litani River, Waremapan rapids; RNH; ♂]. Flint 1996: 419 [distribution]; Holzenthal and Calor 2017: 329 [catalog].

#### Material examined.

Brazil • 1 ♂; Pará, Novo Progresso, REBIO Serra do Cachimbo; 08°44'19.41"S, 54°36'4.81"W; 10 May 2023; light trap; Leandro Juen exped; CZBr 000327.

#### Distribution.

Brazil [new country record] (**Pará**), Peru, Suriname.

### ﻿ Oecetis inflata

Taxon classificationAnimalia

Flint, 1974

A51E339F-D01A-5F4A-A195-28F69F1971C0

#### Literature records.

Flint 1974: 125 [Type locality: Suriname, Wilhelmina Mountains, trail II km 5.7, creek; RNHL; ♂]. Holzenthal and Calor 2017: 337 [catalog].

#### Material examined.

Brazil • 1 ♂; Pará, Novo Progresso, REBIO Serra do Cachimbo, 08°47'28.08"S, 54°31'0.87"W; 21 May 2023; light trap; Leandro Juen exped; CZBr 000328.

#### Distribution.

Brazil [new country record] (**Pará**), Suriname.

##### ﻿Odontoceridae

Odontoceridae is a relatively small family with approximately 180 species distributed in 15 genera (Camargos et al. 2020; Bonfá-Neto et al. 2023). In the Neotropics, Odontoceridae has three genera: *Anastomoneura* Huamantinco & Nessimian, 2004; *Barypenthus* Burmeister, 1839 (both monotypic); and *Marilia* Müller, 1880 (with 48 species) (Bonfá-Neto et al. 2023). Three *Marilia* species have been recorded from Pará (Santos et al. 2025). Here, we present the first record of *Marilia paraguassu* Rocha & Souza, 2018 from the state. In Brazil, this species was previously known only from the state of Maranhão (Rocha and Souza 2018).

### ﻿ Marilia paraguassu

Taxon classificationAnimalia

Rocha & Souza, 2018

1D59C5B6-9C26-5E9E-AD3E-C2E2AD6EE093

#### Literature records.

Rocha and Souza 2018: 397 [type locality: Brazil, Maranhão, Carolina, Parque Nacional Chapada das Mesas, Riacho Estiva, 07°06'59.8"S, 47°21'21.0"W, elev. 265 m, 10–20.x.2013, J.A. Rafael & Limeira-de-Oliveira leg.; DZRJ; ♂].

#### Material examined.

Brazil • 1 ♂; Pará, Barcarena; 01°38'33.07"S, 48°38'59.21"W; 21 Oct. 2022; Leandro Juen exped; CZBr 000329.

#### Distribution.

Brazil (Maranhão, **Pará**).

##### ﻿Polycentropodidae

A high diversity of Polycentropodidae has been reported from the Brazilian Amazon, and new distribution records and descriptions of new species continue to grow (Flint et al. 1999; Chamorro and Holzenthal 2010; Holzenthal and Calor 2017). Among the six extant polycentropodid genera in the Neotropics, five of them are recorded from Brazil; four of these occur in the Brazilian Amazon and three in the state of Pará: *Cernotina* Ross, 1938; *Cyrnellus* Banks, 1913; and *Nyctiophylax* Brauer, 1865. Despite the remarkable diversity of *Polyplectropus*, there were no records from Pará until now. We present the first records of *P. alienus* Bueno-Soria, 1990 and *P. flintorum* Chamorro & Holzenthal, 2010) from Brazil and a new record from Pará of *P. rondoniensis* Chamorro & Holzenthal, 2010.

### ﻿ Polyplectropus alienus

Taxon classificationAnimalia

Bueno-Soria, 1990

07A05E48-8854-5C37-90FB-EE7F4169CF2C

#### Literature records.

Bueno-Soria 1990: 373 [type locality: Mexico, Chiapas, Ocosingo, Finca El Real, Río Santa Cruz; INHS; ♂]; Chamorro and Holzenthal 2010: 47 [diagnosis; ♂; ♀; distribution]; Bueno-Soria and Barba-Álvarez, 2011:361 [checklist]; Holzenthal and Calor 2017: 434 [catalog].

#### Material examined.

Brazil • 2 ♂; Pará, REBIO Serra do Cachimbo; 08°48'10.98"S, 54°32'39.944"W; 23 May 2023; Leandro Juen exped; CZBr 000330.

#### Distribution.

Bolivia, Brazil [new country record] (**Pará**), Mexico.

### ﻿ Polyplectropus flintorum

Taxon classificationAnimalia

Chamorro & Holzenthal, 2010

7FC6AF61-DBD2-55DA-9672-071D67D29958

#### Literature records.

Chamorro and Holzenthal 2010: 89 [type locality: Venezuela, Territorio Federal Amazonas [Estado Amazonas], Camp II, Cerro de la Neblina, 00°50'00"N, 065°59'00"W, elev. 2100 m; NMNH; ♂]. Holzenthal and Calor 2017: 437 [catalog].

#### Material examined.

Brazil • 1 ♂; Pará, Altamira; 03°12'01.74"S, 51°50'28.35"W; 13 Nov. 2023; Leandro Juen exped; CZBr 000331 • 1 ♂; Itaituba, PARNA do Jamanxim; 05°46'13.26"S, 55°44'19.34"W; 31 Jul. 2022; Leandro Juen exped; CZBr 000332 • 1 ♂; same collection data as for preceding; 05°18'26.17"S, 55°56'58.45"W; 20 Jul. 2022; CZBr 000333 .

#### Distribution.

Brazil [new country record] (**Pará**), Venezuela.

### ﻿ Polyplectropus rondoniensis

Taxon classificationAnimalia

Chamorro & Holzenthal, 2010

BE04E343-8F4C-5153-BCDC-3C801AA2793B

#### Literature records.

Chamorro and Holzenthal 2010: 122 [type locality: Brazil, Rondônia, creek 8 km S of Cacaulandia; MZUSP; ♂; ♀]. Paprocki and França 2014: 91 [checklist]; Holzenthal and Calor 2017: 442 [catalog].

#### Material examined.

Brazil • 6 ♂; Pará, Altamira; 03°09'15.0"S, 51°59'01.2"W; 11 Nov. 2023; Leandro Juen exped; CZBr 000334.

#### Distribution.

Brazil (**Pará**, Rondônia).

## Supplementary Material

XML Treatment for Oecetis amplicauda

XML Treatment for Nectopsyche splendida

XML Treatment for Nectopsyche taleola

XML Treatment for Oecetis inflata

XML Treatment for Marilia paraguassu

XML Treatment for Polyplectropus alienus

XML Treatment for Polyplectropus flintorum

XML Treatment for Polyplectropus rondoniensis

## ﻿Additional information

### Author ORCIDs

Fábio B. Quinteiro https://orcid.org/0000-0002-5968-6532

Anne M. Costa https://orcid.org/0000-0002-2138-7596

Gabriel Saldanha https://orcid.org/0009-0000-3157-3387

Laisse Moura https://orcid.org/0009-0006-5122-7637

Otávio T. Assunção https://orcid.org/0009-0004-1615-0684

### Data availability

All of the data that support the findings of this study are available in the main text.
